# Targeting Glycolytic Reprogramming in Gastric Cancer: Navigating the Translational Maze from Mechanism to Clinic

**DOI:** 10.3390/cells15181679

**Published:** 2026-09-16

**Authors:** Guibing Meng, Yulin Li, Limin Gan, Xi Chen, Yitao Chen

**Affiliations:** Jinhua Academy of Zhejiang Chinese Medical University, Jinhua 321000, China; mengguibing@zhezhonglab.ac.cn (G.M.); 202531124011044@zcmu.edu.cn (Y.L.);

**Keywords:** gastric cancer, aerobic glycolysis, Warburg effect, metabolic plasticity, tumor heterogeneity, drug resistance, biomarkers, combination therapy, immunotherapy, precision metabolic medicine

## Abstract

Gastric cancer (GC) remains one of the leading causes of cancer-related mortality worldwide. Among its defining hallmarks, metabolic reprogramming, particularly aerobic glycolysis (the Warburg effect), emerged as a central driver of tumor progression, therapeutic resistance, and immune evasion. Over the past decades, substantial efforts have delineated the molecular architecture of metabolic reprogramming in GC, identifying key effector enzymes, including hexokinase 2 (HK2), pyruvate kinase M2 (PKM2), and lactate dehydrogenase A (LDHA), as well as upstream oncogenic signaling axes, such as the PI3K/AKT/mTOR pathway and hypoxia-inducible factor 1-alpha (HIF-1α), along with their interconnected regulatory networks. Despite extensive preclinical validation of these nodes, clinical translation remains elusive, with glycolysis-targeted monotherapies showing limited efficacy in early-phase trials. Yet, we contend that this persistent translational failure does not stem from invalid targets, but rather from a systemic underestimation of three fundamental roadblocks: (1) temporal metabolic plasticity that enables rapid compensatory adaptation and pathway switching; (2) spatial inter- and intra-tumoral metabolic heterogeneity that undermines uniform treatment strategies; and (3) a critical void in predictive and pharmacodynamic biomarkers essential for patient stratification and treatment monitoring. To overcome these barriers, we propose an integrated, forward-looking strategic framework that converges advanced diagnostics with next-generation therapeutic modalities. On the diagnostic front, we highlight spatial multi-omics for high-resolution metabolic cartography and artificial intelligence-driven integrative patient stratification to map heterogeneity and predict treatment response. On the therapeutic front, we examine strategies designed to circumvent metabolic plasticity, including dual-pathway inhibition, nodal targeting, exploitation of non-catalytic vulnerabilities, and tumor-penetrating nanocarriers for targeted metabolic intervention. Particular emphasis is placed on rational, mechanism-driven combination regimens, especially those synergizing glycolysis-targeted therapies with immunotherapy to remodel the suppressive tumor microenvironment—as well as hierarchical and parallel pathway combinations and the emerging metabolism–epigenetics axis, exemplified by lactate-mediated histone lactylation and α-ketoglutarate-dependent DNA demethylation. By shifting the therapeutic paradigm from static inhibition of single metabolic nodes toward dynamic, network-level intervention, this review provides a strategic roadmap for translating the vulnerabilities inherent in the glycolytic network into durable clinical benefit for patients with GC. This paradigm shift, we argue, is essential for advancing precision metabolic medicine beyond the current impasse.

## 1. Introduction

Gastric cancer (GC) is the fifth leading cause of cancer-related mortality globally [1], with China contributing 70% and 50% of new cases of cardia and non-cardia GC, respectively [2]. The incidence of GC in China was reported at 25.41 per 100,000 people in 2022 [3]. Current treatment strategies for GC now encompass a comprehensive framework integrating diagnostic assessment, neoadjuvant therapy, adjuvant surgical resection, and, in selected cases, precisely targeted radiotherapy. Endoscopic examination and forceps biopsy are the gold standard methods for diagnosing gastric cancer [4]. The MATTERHORN regimen—perioperative durvalumab combined with FLOT chemotherapy—has emerged as a promising new standard of care for locally advanced gastric cancer [5]. Beyond systemic therapy, the extent of lymph node dissection in gastric cancer surgery has remained a subject of ongoing debate; recent evidence suggests that disease-specific lymphadenectomy may improve outcomes and represents an important consideration in surgical decision-making [6]. Although these approaches can modestly improve patient survival, they are still associated with substantial limitations, including limited efficacy in advanced stages, significant systemic toxicity, and high rates of recurrence and metastasis. Therefore, the identification of novel therapeutic targets and the development of innovative treatment strategies are critical priorities in contemporary GC research. Among emerging targets, metabolic reprogramming garners particular attention due to its central role in tumor adaptation and therapeutic resistance.

Metabolic reprogramming is widely recognized as a hallmark of malignant tumors [7]. It involves metabolic changes that enable cancer cells to acquire the energy needed for survival and rapid proliferation. One notable example is the Warburg effect [8], in which tumor cells preferentially utilize glycolysis over mitochondrial respiration for energy production despite the presence of adequate oxygen, thereby generating not only ATP but also metabolic intermediates essential for biosynthesis of nucleotides, lipids, and amino acids. In GC, this glycolytic shift is particularly pronounced and is linked to tumor initiation, progression, and resistance to conventional therapies.

This metabolic dependence propels glycolytic pathways to the forefront of anticancer drug development. Extensive mechanistic studies have identified a core set of dysregulated effectors in GC, including rate-limiting enzymes such as hexokinase 2 (HK2), pyruvate kinase M2 (PKM2), and lactate dehydrogenase A (LDHA), whose overexpression consistently correlates with aggressive disease and poor prognosis [9,10,11]. These enzymes are embedded within oncogenic signaling circuits, most notably the PI3K/AKT/mTOR axis and hypoxia-inducible factor 1-alpha (HIF-1α), with HIF-1α serving as a key downstream transcriptional effector that integrates microenvironmental hypoxia with growth signals to enforce a glycolytic phenotype [12,13,14,15]. Accordingly, a wide range of preclinical studies employing small-molecule inhibitors, genetic perturbations, or natural compounds demonstrated robust anti-tumor effects in vitro and in vivo [15,16,17,18]. Yet, whether these preclinical successes can be replicated in the clinical setting remains an open question.

Paradoxically, this compelling preclinical narrative does not translate into clinical success. To date, no glycolysis-targeted therapy has achieved regulatory approval as a monotherapy for GC, despite decades of intensive investigation, and this persistent failure highlights the challenge of translating preclinical findings into clinical benefit [19]. In this context, the study of metabolic reprogramming in GC evolves from a mechanistic understanding of metabolic alterations into a translational research frontier demanding integrated diagnostic–therapeutic strategies [20].

**Figure 1:**
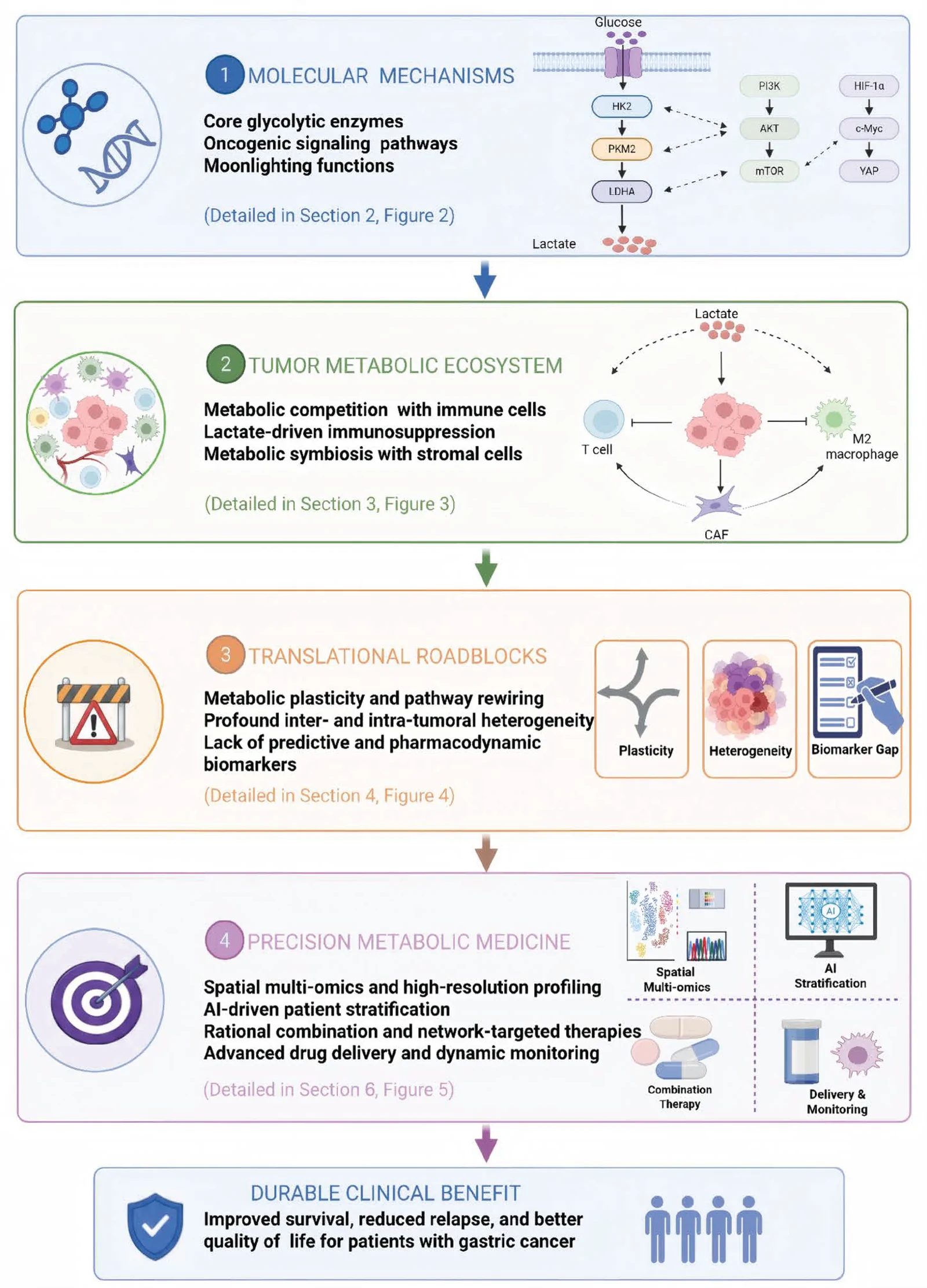
Conceptual roadmap of metabolic reprogramming and precision metabolic medicine in GC. This review follows a translational framework from mechanistic understanding to clinical application. Metabolic reprogramming is driven by dysregulated enzymes, oncogenic signaling pathways, and moonlighting functions. These metabolic alterations reshape the tumor ecosystem through metabolic competition, lactate signaling, and stromal interactions. However, clinical translation is hindered by three major barriers, including metabolic plasticity, tumor heterogeneity, and the lack of predictive biomarkers. Finally, emerging strategies such as spatial multi-omics, artificial intelligence-assisted stratification, rational combination therapies, and precision metabolic medicine are discussed as future directions to overcome these challenges.

This review focuses on the translational dimension of metabolic reprogramming, moving beyond mechanistic cataloging. We first delineate the molecular architecture of metabolic reprogramming in GC, focusing on the core enzymes, regulatory circuits, and network properties that create both therapeutic vulnerabilities and adaptive resilience. We then systematically dissect three interrelated translational roadblocks that impede clinical success: adaptive metabolic plasticity, profound metabolic heterogeneity, and a critical void in predictive biomarkers. Finally, we synthesize an integrated diagnostic–therapeutic framework designed to overcome these barriers. By bridging mechanistic insight with emerging diagnostic and therapeutic innovations, we seek to provide a strategic guide for accelerating the clinical translation of metabolism-targeted therapy in GC (Figure 1).

## 2. The Glycolytic Network in GC: A Foundation of Vulnerability and Resilience

To appreciate why glycolysis represents both an attractive therapeutic target and a recurrent translational failure, it is essential to briefly outline the architecture of the glycolytic network in GC. This network is not a linear metabolic pathway but a highly regulated, multi-layered system characterized by redundancy, feedback control, and extensive crosstalk with oncogenic signaling and other metabolic routes that integrate cellular energy demands. These features simultaneously create metabolic vulnerabilities such as enzyme isoform dependency and dependence on high glycolytic flux and confer remarkable resilience to therapeutic perturbation through compensatory mechanisms. The following sections detail the core enzymes, upstream signaling axes, network integration, and noncatalytic moonlighting functions that together constitute this foundation.

### 2.1. Core Effector Enzymes: Drivers of the Glycolytic Flux

The glycolytic flux in GC is driven by multiple rate-limiting enzymes, including HK2/HKDC1, PKM2, LDHA, ENO1, and PGK1, whose dysregulation contributes to tumor progression and poor prognosis (Table 1).

#### 2.1.1. HK2 and HKDC1

Hexokinases (HKs) catalyze the phosphorylation of glucose to G6P, which is the first step of glycolysis and the key rate-limiting step that determines metabolic flux. The aberrant activation of HK2 and the novel isozyme hexokinase domain-containing 1 (HKDC1) constitutes the core of tumor metabolic reprogramming in GC.

The high expression of HK2 is a consequence of coevolution in GC. In addition to the classical PI3K/AKT/mTOR pathway driving its expression, the inflammation-related protein NLRP12, through competitive binding to TRIM25, blocks TRIM25-mediated ubiquitination and degradation of HK2 in GC, thereby maintaining a high level of glycolytic activity via post-translational stabilization [21]. Furthermore, spalt-like transcription factor 4 (SALL4) transcriptionally activates HK2 expression, enhances glycolytic metabolism, and consequently promotes the malignant phenotype of GC cells [22].

The increased rate of glucose phosphorylation mediated by HK2 directly enhances glycolytic flux. More importantly, HK2 possesses a unique intracellular localization. It binds to the voltage-dependent anion channel (VDAC) on the outer mitochondrial membrane via its N-terminal hydrophobic domain. This localization allows HK2 to preferentially utilize mitochondrial ATP for phosphorylation and to reduce cell damage caused by reactive oxygen species (ROS) generated from oxidative phosphorylation (OXPHOS) [21,22].

This metabolic coupling directly blocks the translocation of pro-apoptotic proteins to the mitochondria, thereby establishing an anti-apoptotic barrier for GC cells that enhances their survival under chemotherapeutic stress [21,22].

Retrospective clinical cohort studies have shown that HK2 expression levels are significantly positively correlated with TNM stage and lymph node metastasis in GC [23]. However, the existence of HKDC1 as a compensatory isozyme, capable of sustaining glycolysis when HK2 is limited or inhibited, illustrates the inherent redundancy of the glycolytic network.

Notably, the complexity of GC metabolism extends beyond the activation of PI3K/AKT-driven HK2 activation. Recent studies have revealed that even when HK2 expression is limited or selectively inhibited, GC cells can still maintain extremely high glycolytic rates. This is largely attributable to another member of the hexokinase family-HKDC1. Unlike HK2, the activation of HKDC1 in GC exhibits pronounced environmental dependence. Studies showed that *Helicobacter pylori* (*H. pylori*), a major risk factor for GC, can directly induce HKDC1 expression through activation of the TGF-β/Smad2 signaling pathway. This finding effectively links chronic inflammation to metabolic reprogramming [24,42]. Furthermore, in GC subtypes with low HK2 expression, HKDC1 sustains basal glycolytic efficiency and ensures energy homeostasis, reflecting the robustness of the metabolic network. The introduction of HKDC1 enhances the metabolic plasticity of GC cells under hypoxic or nutrient-deprived conditions [25,43,44]. Of note, the role of HKDC1 extends well beyond metabolic regulation [25]. It is identified as a key driver of epithelial–mesenchymal transition (EMT), significantly enhancing the migratory capacity and invasive potential of GC cells into the surrounding stroma through the upregulation of transcription factors such as Snail. High HKDC1 expression serves as an independent risk factor for poor prognosis in GC, and notably, in patients with *H. pylori*-positive diffuse-type GC, HKDC1 exhibits superior prognostic biomarker value compared to conventional HK2 [24].

#### 2.1.2. PKM2

At the terminal step of the glycolytic flux in GC, PKM2 acts as the key rate-limiting enzyme that catalyzes the conversion of phosphoenolpyruvate (PEP) to pyruvate. Unlike the aforementioned HKs, the pro-tumorigenic role of PKM2 arises not only from the upregulation of its expression level, but also from its conformational switching between the high-activity tetramer and the low-activity dimer, a property not shared by other glycolytic rate-limiting enzymes.

Studies showed that Rab25, a member of the small GTPase family, exerts a significant pro-tumorigenic role in gastric adenocarcinoma by interacting with PKM2 to regulate aerobic glycolysis, thereby providing metabolic power for tumor growth [26]. Furthermore, the epigenetic regulator KAT2A was demonstrated to promote succinylation of PKM2, a novel modification that serves as a critical switch for the precise switching of metabolic modes in GC cells [27].

When PKM2 exists in its low-activity dimeric form, the terminal flux of glycolysis is blocked, leading to the accumulation of upstream metabolic intermediates. In response to this alteration, the accumulated substrates are forced to be shunted into the pentose phosphate pathway (PPP) and the amino acid synthesis pathway [45]. This metabolic reprogramming is particularly important for GC, as it provides the necessary nucleotide precursors for rapid cancer cell division and the reducing power NADPH for ROS scavenging, rather than merely generating ATP. Beyond its metabolic role, PKM2 also exhibits moonlighting functions in the nucleus.

#### 2.1.3. LDHA

LDHA catalyzes the final step of glycolysis, reducing pyruvate to lactate while simultaneously regenerating NAD^+^ from NADH [46]. The primary bioenergetic value of LDHA lies in this cofactor regeneration effect. By rapidly replenishing the NAD^+^ pool, LDHA meets the critical co-factor demands of upstream glyceraldehyde-3-phosphate dehydrogenase (GAPDH), thereby preventing metabolic stagnation even under hypoxic conditions. This ensures a high-velocity glycolytic flux that provides both ATP and biosynthetic intermediates. While the metabolic role of LDHA is central to intracellular homeostasis, the extrinsic effects of its byproduct, lactate, act as a signaling messenger and modulator of the tumor microenvironment (TME).

#### 2.1.4. Other Key Glycolytic Enzymes

Additional enzymes further reinforce metabolic reprogramming in GC. Enolase 1 (ENO1) promotes tumor proliferation, metastasis, stemness, and cisplatin resistance, frequently through activation of AKT signaling and regulation by non-coding RNAs (ncRNAs) or SUMOylation [32,33,34]. Phosphoglycerate kinase 1 (PGK1) is subject to opposing ubiquitin-mediated regulation, with TRIM8 promoting its stabilization and glycolysis, whereas TRIM50 facilitates its degradation to suppress metabolic flux [35,36]. Pyruvate dehydrogenase kinases (PDKs), particularly PDK4, inhibit pyruvate entry into the TCA cycle, thereby diverting pyruvate toward lactate synthesis [39]. In contrast, aldolase B (ALDOB) often functions as a tumor suppressor, and its downregulation enhances glycolysis through activation of the PI3K/AKT pathway [40,41].

Collectively, these enzymes form a tightly interconnected and redundantly regulated glycolytic machinery, ensuring robust metabolic output even under fluctuating environmental conditions.

### 2.2. Regulatory Architecture: Signaling Axes and Oncogenic Networks Overriding Metabolism

#### 2.2.1. PI3K/AKT/mTOR and HIF-1α Signaling Axes

The PI3K/AKT/mTOR axis and HIF-1α constitute a central signaling hub that drives aerobic glycolysis in GC [47,48,49]. The coupling of these two axes not only integrates extracellular environmental stress but also maintains the tumor’s metabolic phenotype through complex feedback loops.

The metabolic transformation of GC exhibits significant pathogen dependence and genetic heterogeneity. *H. pylori* infection is a key inducing factor that triggers metabolic transformation of the gastric mucosa. Studies have shown that CagA, upon tyrosine phosphorylation by host Src family kinases, can directly activate the PI3K/AKT pathway, thereby inducing HIF-1α expression under normoxic conditions [50]. Furthermore, CagA can localize to the mitochondria and, through inhibition of SIRT3, lead to elevated ROS levels, further enhancing the stability and activity of HIF-1α [51]. Although current research on glycolysis has largely focused on signal transduction, large-scale genomic studies have confirmed the presence of high-frequency PIK3CA mutations in GC, which provide a potential molecular basis for the constitutive activation of the PI3K pathway, while the direct causal link to glycolytic flux remains to be functionally validated.

Activation of the PI3K/mTOR pathway is a key mechanism underlying the upregulation of HIF-1α under normoxic conditions. This regulation is primarily achieved by enhancing HIF-1α protein stability via inhibition of proteasomal degradation, enabling GC cells to exhibit a pronounced Warburg effect even in areas with abundant blood supply. The sustained activation of this signaling axis is not unidirectional but is self-reinforced through complex regulatory loops. For example, a HIF-1α/miR-520a-3p/AKT1/mTOR feedback axis was demonstrated in GC: HIF-1α suppresses the expression of miR-520a-3p, and loss of miR-520a-3p derepresses AKT1, thereby further promoting HIF-1α protein accumulation via mTOR [52].

Activated HIF-1α cooperatively upregulates multiple metabolism-related genes, including glucose transporter 1 (GLUT1), by binding to the hypoxia response elements (HREs) in the promoter regions of target genes [11,12,13,14]. GLUT1 was shown to be involved in the uptake of glucose into cells.

Activation of this signaling axis not only drives tumor growth but also confers significant stress tolerance capabilities on cells, as *H. pylori* infection is closely associated with drug resistance through Akt-mediated metabolic reprogramming [53]. Notably, although the CagA-induced increase in ROS serves as an initial activation signal, the subsequent enhancement of glycolysis may modulate redox balance by generating reducing equivalents, thereby helping tumor cells resist lethal oxidative stress induced by 5-FU or cisplatin. Interventions targeting HIF-1α can not only inhibit *H. pylori*-mediated microbial virulence but also effectively attenuate the host inflammatory response and reverse the drug-resistant phenotype [54].

#### 2.2.2. Therapy Resistance and Post-Transcriptional Regulation

During the metabolic progression of GC, post-transcriptional regulatory mechanisms serve as a regulatory means at the non-genomic level, conferring significant metabolic plasticity on tumor cells by modulating mRNA stability independently of gene copy number variations.

GC cells establish an efficient metabolic fine-tuning mechanism at the post-transcriptional level through high expression of the RNA-binding protein insulin-like growth factor 2 mRNA-binding protein 2 (IGF2BP2). As a typical N^6^-methyladenosine (m^6^A) reader protein, IGF2BP2 specifically recognizes and binds to m^6^A modification sites on target mRNAs [55]. Studies confirmed that in GC, IGF2BP2 significantly prolongs the intracellular half-life and increases the protein expression abundance of HIF-1α by recognizing and stabilizing its mRNA, particularly the “GGACU” motif within its coding region [56]. This regulatory mode constitutes an important non-transcriptional contribution to HIF-1α accumulation, enabling HIF-1α to maintain high levels of biological activity under stress conditions.

The steady-state maintenance of HIF-1α mRNA by IGF2BP2 directly drives metabolic reprogramming in GC cells. Experimental evidence shows that high expression of IGF2BP2 significantly enhances the glycolytic activity of GC cells, a process that primarily depends on its positive regulation of HIF-1α expression [56]. This IGF2BP2-mediated metabolic enhancement effect was reported in various malignant tumors including glioblastoma [57], such as glioblastoma, indicating that this post-transcriptional regulatory axis represents a universal mechanism by which tumors maintain a high glycolysis phenotype. IGF2BP2-mediated metabolic reprogramming is one of the core factors contributing to radiotherapy resistance in GC. Studies confirmed that knockdown of IGF2BP2 significantly exacerbates radiation-induced ROS burst and DNA damage, thereby enhancing the radiosensitivity of cells [56].

IGF2BP2, as an important molecular barrier to radiotherapy resistance, provides a new dimension for the prognostic assessment of GC. Given that this mechanism involves the regulation of RNA stability at the non-genomic level, small-molecule interventions targeting IGF2BP2 or its binding sites with HIF-1α hold promise as a prospective therapeutic strategy to reverse acquired radiotherapy resistance by attenuating the metabolic resilience of tumor cells under radiation stress.

### 2.3. An Integrated and Flexible Metabolic Network

Beyond its regulation by upstream signaling axes, the glycolytic network in GC exhibits emergent properties that enable tumor cells to thrive under fluctuating microenvironmental conditions. This network is not a linear catabolic pathway but a high-flux metabolic hub, intricately integrated into a multi-dimensional system that orchestrates tumor resilience.

#### 2.3.1. Branching Pathways and the Hybrid Metabolic Phenotype

Glycolytic flux is strategically shunted into crucial branching pathways. The PPP is preferentially activated to sustain redox homeostasis via NADPH production and to provide ribose-5-phosphate for accelerated nucleotide synthesis [58]. Simultaneously, GC cells exhibit a hybrid metabolic phenotype, wherein glycolytic intermediates are coupled with mitochondrial OXPHOS and an intensified glutaminolysis to meet the bioenergetic and biosynthetic demands of rapid proliferation [59,60].

#### 2.3.2. NcRNA Regulation

This metabolic architecture is further refined by a sophisticated regulatory layer comprising ncRNAs, including long non-coding RNAs (lncRNAs) and circular RNAs (circRNAs), which act as regulators for glycolytic enzymes through competing endogenous RNA (ceRNA) networks or direct protein-binding [61,62,63].

#### 2.3.3. Cross-Pathway Crosstalk in the Metabolic Network

Importantly, the signaling pathways that regulate glycolysis do not operate in isolation but converge at key metabolic nodes. The β-catenin/c-Myc axis, activated by upstream factors such as ZNF479, transcriptionally upregulates the glycolytic enzyme repertoire to provide the material and energy basis for rapid tumor proliferation [64]. Concurrently, the Hippo/YAP pathway, by sensing mechanical stress from the microenvironment, induces YAP nuclear translocation, which not only enhances glycolytic flux but also coordinately upregulates lactate efflux transporters, effectively maintaining intracellular acid-base homeostasis under high metabolic rates [65,66]. At the level of energy surveillance, the AMPK/mTOR axis serves as a critical metabolic checkpoint, finely tuning metabolic output by sensing energy load; pharmacological activation of AMPK using agents such as metformin can effectively disrupt the metabolic homeostasis of GC cells and suppress their growth potential [67,68].

These signaling pathways broadly converge at key regulatory nodes such as mTOR and PKM2 and STAT3/c-Myc. This cross-pathway synergistic effect endows GC cells with remarkable metabolic plasticity and simultaneously constitutes the molecular basis for the propensity of single-pathway blockade therapies to develop acquired resistance in clinical settings [69].

#### 2.3.4. Network Resilience as a Therapeutic Barrier

Such extensive interconnectivity and compensatory plasticity allow GC cells to thrive under the fluctuating stressors of the TME. Consequently, the robust redundancy of this network constitutes a formidable barrier to monotherapy. Pharmacological inhibition of a single metabolic node is frequently circumvented by rapid flux redistribution, which underscores the necessity of shifting from node-targeted to network-level therapeutic strategies. These properties of flexibility and redundancy are schematically summarized and form the conceptual foundation.

### 2.4. Moonlighting Functions: Glycolytic Enzymes as Master Regulators of Cell Fate

**Figure 2:**
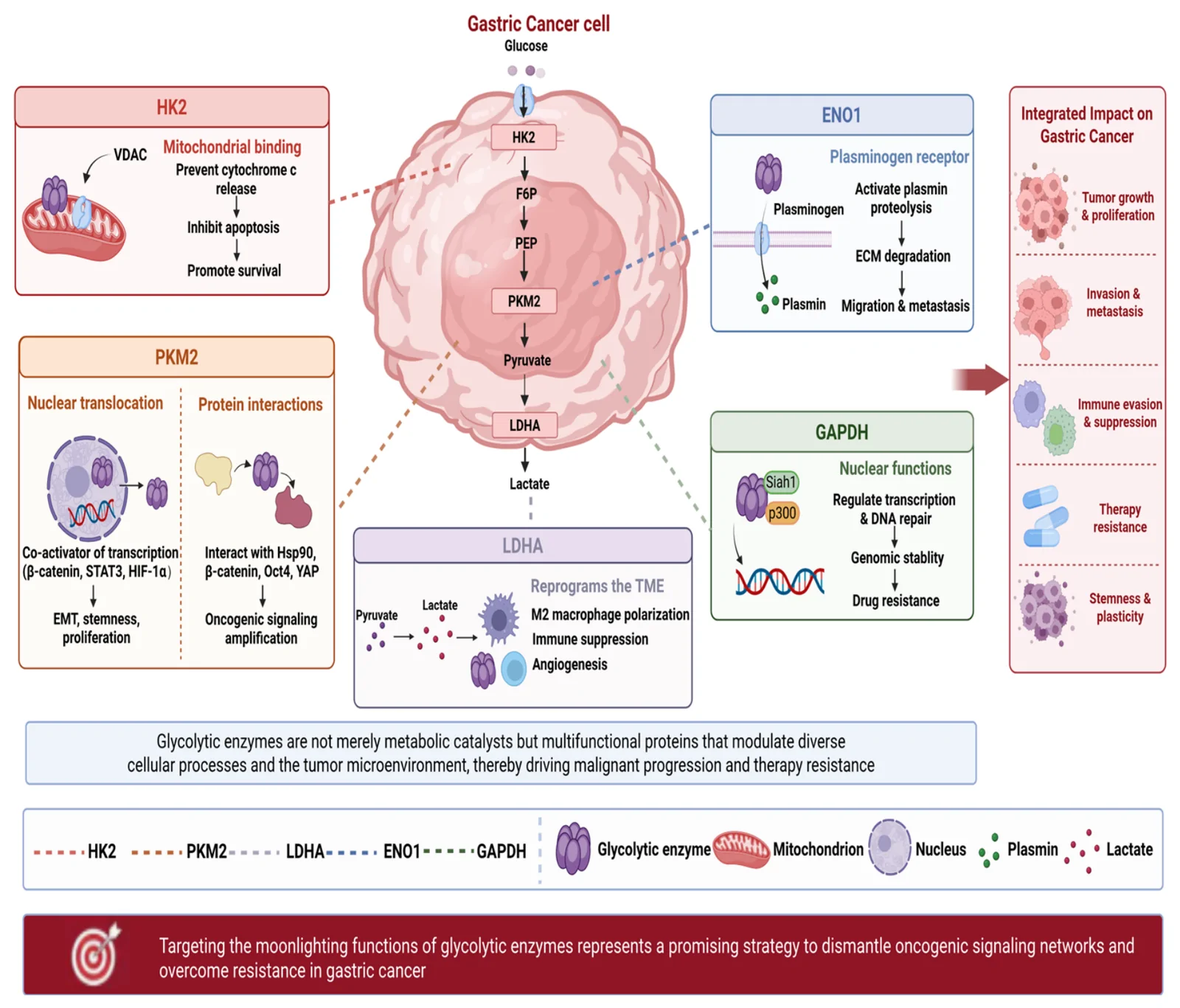
Moonlighting functions of glycolytic enzymes in GC. Beyond their canonical metabolic roles, glycolytic enzymes acquire diverse non-catalytic functions through altered subcellular localization and protein–protein interactions. HK2 promotes cell survival via mitochondrial VDAC binding; PKM2 functions as a nuclear transcriptional co-activator; ENO1 facilitates invasion through plasminogen activation; GAPDH participates in transcriptional regulation and DNA repair; and LDHA-derived lactate reshapes the TME. Collectively, these moonlighting activities contribute to tumor progression, immune evasion, stemness maintenance, and therapeutic resistance.

Beyond their canonical catalytic roles in energy metabolism, glycolytic enzymes in GC exhibit remarkable moonlighting functions. Upon induction by specific stresses, these proteins adopt transcriptional co-factors, protein kinases, or signaling scaffolds through alterations in subcellular localization or post-translational modifications (PTMs) [70,71]. This functional switching directly couples the cellular metabolic state to gene expression and survival decisions, constituting the underlying molecular mechanism of malignant progression in GC [72,73] (Figure 2).

#### 2.4.1. Nuclear Translocation: Orchestrating the Transcriptional Landscape

GC cells achieve direct communication between metabolic signals and nuclear genetic programs by precisely translocating cytoplasmic glycolytic enzymes into the nucleus [74]. This nuclear moonlighting function not only provides substrates required for epigenetic modifications but also directly alters oncogene expression by regulating transcriptional complexes [28,75]. As a typical representative of moonlighting functions, the nuclear translocation of dimeric PKM2 serves as a critical switch for the malignant progression of GC [76]. Upon stimulation by growth factor signals such as human epidermal growth factor receptor 2 (HER2), ERK1/2 phosphorylates PKM2 at Ser37, which subsequently recruits the peptidyl-prolyl cis-trans isomerase PIN1 to induce a conformational change, exposing the nuclear localization sequence (NLS) and facilitating its translocation into the nucleus. Once in the nucleus, PKM2 exhibits minimal catalytic activity but instead functions as a transcriptional coactivator by binding to β-catenin, and in GC cell line models, PKM2 has been reported to promote the expression of CCND1 and MYC, thereby driving cell cycle progression []. Furthermore, nuclear PKM2 also activates the STAT3 signaling pathway, induces EMT, and confers acquired resistance to 5-FU in GC cells [29,77].

GAPDH exhibits a strong redox sensing capability in GC. Under oxidative stress conditions, GAPDH undergoes S-nitrosylation (SNO-GAPDH), subsequently forms a complex with the E3 ubiquitin ligase SIAH1, and translocates into the nucleus [78]. Nuclear GAPDH, by stabilizing the mRNA of specific oncogenes or modulating p53 activity, establishes an anti-apoptotic program under metabolic stress, significantly enhancing the survival fitness of GC cells [79].

#### 2.4.2. Non-Canonical Kinase Activity: Organelle Hijacking and Epigenetic Remodeling

Studies revealed that the glycolytic kinase PDK1 can utilize metabolic intermediates as phosphate donors to phosphorylate non-metabolic protein substrates, thereby blocking apoptotic signals or directly modifying the epigenetic landscape [80]. PGK1 exhibits unique inter-organelle regulatory functions in GC. Upon activation by hypoxia or oncogenic signaling, PGK1 is phosphorylated at Ser203 and translocates to the mitochondria. Within the mitochondria, PGK1 acts as a protein kinase, phosphorylating PDK1 at Thr338, thereby inhibiting the activity of the pyruvate dehydrogenase (PDH) complex [37]. This mechanism not only enhances the Warburg effect but also confers anoikis resistance on GC cells during peritoneal dissemination by inhibiting mitochondrial ROS production [38]. Beyond its role as a transcriptional coactivator (Section 2.4.1), nuclear PKM2 can also directly phosphorylate histone H3 at the Thr11 residue [28]. This epigenetic mark leads to chromatin remodeling and specifically upregulates genes such as SLC2A1, forming a positive feedback loop between metabolic reprogramming and gene regulation. This role transition from metabolite producer to epigenetic mark sculptor represents a core pathway through which GC cells achieve long-term adaptive evolution [28].

#### 2.4.3. Molecular Scaffolding: Integrating Oncogenic Signaling Chains

Glycolytic enzymes act as molecular scaffolds through protein–protein interactions (PPIs), integrating and amplifying oncogenic signals in GC [81]. ENO1 serves as a paradigmatic example of moonlighting in glycolytic enzymes. In GC, surface-localized ENO1 recruits plasminogen and facilitates extracellular matrix degradation. Cytoplasmic ENO1, however, serves as a scaffolding protein for the PI3K/AKT signaling axis. Through SUMO modification, this cytoplasmic pool increases signal transduction efficiency. These effects ultimately promote tumor invasion and cisplatin resistance [82]. Aldolase A (ALDOA) serves as a glucose sensor on the lysosomal surface. When glucose levels fluctuate, ALDOA facilitates the assembly of the V-ATPase complex, thereby precisely regulating mTORC1 activity [81]. This scaffolding function ensures that the protein synthesis rate of GC cells is synchronized in real time with environmental nutrient abundance.

#### 2.4.4. Therapeutic Implications: From Catalytic Inhibition to Moonlighting Blockade

This multi-identity switching of glycolytic enzymes is a major source of acquired resistance in GC. Traditional metabolic inhibitors often only block the catalytic site but fail to interfere with enzyme nuclear translocation or kinase activity [83]. Therefore, developing blocking peptides targeting GC-specific PTM sites, or employing proteolysis-targeting chimeras (PROTAC) technology for total protein degradation, represents a new frontier in precision metabolic therapy [81,84].

In summary, the glycolytic network in GC is characterized by three key features that simultaneously create therapeutic vulnerabilities and confer intrinsic resilience:

(1) Enzymatic redundancy. The presence of compensatory isozymes and metabolic bypasses ensures that glycolysis can be sustained even when a single node is inhibited.
(2) Regulatory interconnectivity. Extensive crosstalk among upstream signaling axes and post-transcriptional regulation creates a highly robust control system that resists pathway-specific blockade.
(3) Functional multitasking. Moonlighting functions of glycolytic enzymes drive oncogenic programs independently of their catalytic roles, creating a pharmacological blind spot for conventional active-site inhibitors.

These very features—while making glycolysis an attractive therapeutic target—also render the network remarkably resistant to single-agent inhibition. This understanding provides a foundation for the following discussion of the tumor ecosystem (Section 3) and the three translational roadblocks (Section 4). However, the influence of metabolic reprogramming extends far beyond the cancer cell itself. Through metabolic competition, lactate signaling, and niche construction, GC cells actively remodel their surrounding microenvironment, creating an ecosystem that fuels tumor progression and therapeutic resistance. The following section dissects this glycolytic ecosystem in detail.

## 3. The Glycolytic Ecosystem in the TME

The metabolic progression of GC is not an isolated intracellular event but rather an ecological remodeling of TME driven by glycolytic flux. Through intensified glycolysis, GC cells not only meet their own biomass demands but also, by depleting limited resources and releasing metabolic byproducts, construct a niche characterized by high acidity, hypoxia, and immunosuppression. Understanding this ecosystem is essential, as the microenvironmental alterations, especially metabolic competition and lactate signaling, directly contribute to translational obstacles in therapeutic resistance and immunotherapy failure.

**Figure 3:**
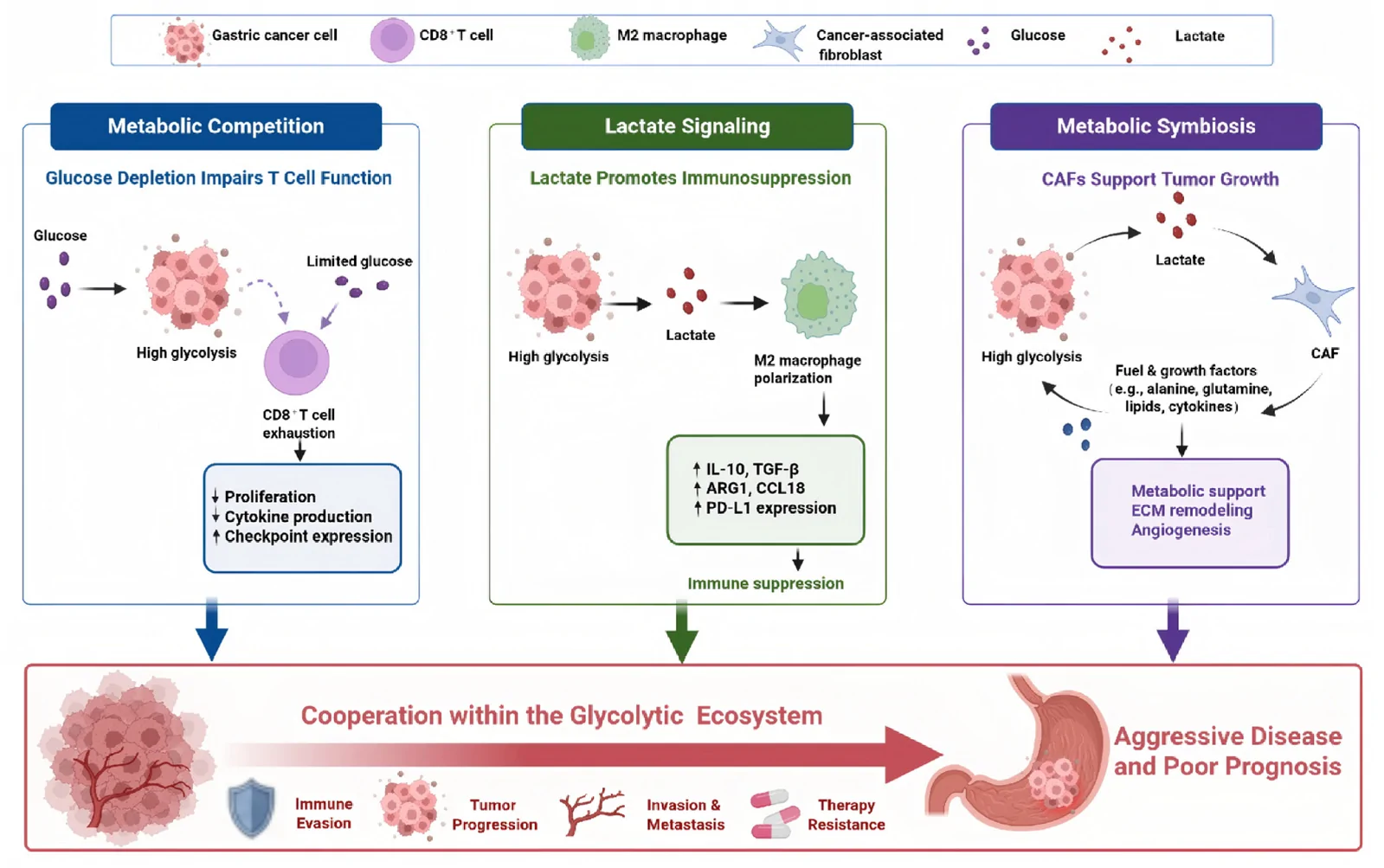
The glycolytic ecosystem within the TME of GC. Glycolytic GC cells consume large amounts of glucose and secrete lactate, thereby reshaping the TME through metabolic competition and intercellular signaling. Lactate suppresses CD8^+^ T-cell function, promotes M2 macrophage polarization, supports cancer-associated fibroblast (CAF)-mediated metabolic symbiosis, and contributes to the establishment of an immunosuppressive niche that facilitates tumor progression.

This section focuses on three key aspects of the glycolysis ecosystem: metabolic competition with immune cells, lactate-driven signaling and epigenetic reprogramming, and the unique niche construction driven by *H. pylori* and the acidic microenvironment (Figure 3).

### 3.1. Metabolic Competition: Resource Scarcity and Immune Exhaustion

Within the confined ecosystem of the TME, a fierce resource battle exists between GC cells and infiltrating immune cells. GC cells, through marked upregulation of GLUT1/3 and HK2, exert a powerful glucose consumption effect on the local interstitial space. Since the activation of effector T cells is highly dependent on glycolysis, substrate deprivation forces T cells into a state of metabolic starvation, directly resulting in impaired activation of their endogenous mTORC1 pathway. This metabolic restriction significantly reduces the secretion of IFN-γ and granzyme B by T cells, inducing them into a state of functional exhaustion [85,86].

Lactic acid, the end product of glycolysis, further amplifies immunosuppression [87]. Studies confirmed that lactate, via the MCT-HIF1α signaling axis, induces polarization of tumor-associated macrophages (TAMs) toward the M2 phenotype and upregulates PD-L1 expression [30,31]. This metabolic competition-driven immune evasion is a major contributor to immunotherapy resistance in GC [88].

### 3.2. Lactate Signaling: Driving Invasiveness and Epigenetic Reprogramming

Lactate transforms from a metabolic byproduct into a highly bioactive signaling molecule, dominating the malignant progression of GC through non-canonical signaling and epigenetic modifications.

Beyond its conventional metabolic catalytic function, LDHA exhibits a remarkable non-canonical mechanism: in experimental models, LDHA has been shown to activate Rac1 GTPase in an enzyme-independent manner, inducing cytoskeletal remodeling and promoting cell migration [89].

Lactate generated from glycolysis also acts as a donor to mediate Kla of histones, thereby initiating a pathway from glucose uptake to LDHA-mediated Kla [10]. In GC, clinical correlative studies have shown that H3K18la levels are associated with poor prognosis and preclinical evidence indicates that they are dynamically regulated by SIRT1-mediated delactylation [90,91].

Furthermore, lactate-induced histone lactylation is integrated into the global framework of microbe–host interactions [92], and lactylation sites on non-histone proteins in GC cells number over 1000, indicating that this modification is integrated into the global framework of microbe–host interactions.

### 3.3. Niche Construction: H. pylori and Acidic Orchestration

The unique etiological background of GC and its anatomical environment endow its glycolytic ecosystem with unique complexity. As a group I carcinogen, *H. pylori* stabilizes HIF-1α in host cells through its virulence factor CagA, forcibly initiating a pseudo-hypoxic response even under normoxic conditions. This process significantly enhances host glycolytic flux [44], and the lactate produced further augments the invasive phenotype of cancer cells by inducing histone lactylation [90].

To cope with the dual pressure of gastric acid and lactate efflux, GC cells are highly dependent on carbonic anhydrase IX (CA IX) to maintain pH homeostasis. CA IX not only exacerbates interstitial acidification to suppress immunity but also generates an ion-trapping effect, leading to the extracellular polarization of weakly basic chemotherapeutic agents, thereby establishing a metabolism-driven physical barrier to drug resistance [86,93].

In summary, the glycolytic ecosystem in GC extends far beyond the cancer cell itself. Through metabolic competition, lactate signaling, and niche construction, GC cells actively remodel their microenvironment to suppress antitumor immunity, drive invasive progression, and establish physical barriers to chemotherapy. These microenvironmental alterations, metabolic competition, lactate-driven immune suppression, and pH-mediated drug resistance, are not isolated phenomena. Together with the network properties described in Section 2, they converge into three major translational roadblocks that consistently impede clinical success. The following section systematically deconstructs these roadblocks.

## 4. Deconstructing the Translational Roadblocks

Metabolic reprogramming is a potent driver of malignant progression. This effect stems from its regulatory complexity, redundancy, and extensive network integration. Nevertheless, these features also pose the primary obstacles to effective therapeutic targeting. As introduced in Section 1 and foreshadowed in the summary above, three interrelated translational roadblocks emerge as dominant determinants of therapeutic failure:

(1) Profound metabolic heterogeneity across scales, which undermines uniform treatment strategies;
(2) Adaptive metabolic plasticity, including pathway compensation, feedback activation, and moonlighting-driven resistance, which enables rapid therapeutic escape;
(3) A critical void in predictive and pharmacodynamic biomarkers, which impedes patient stratification and real-time monitoring of target engagement.

**Figure 4:**
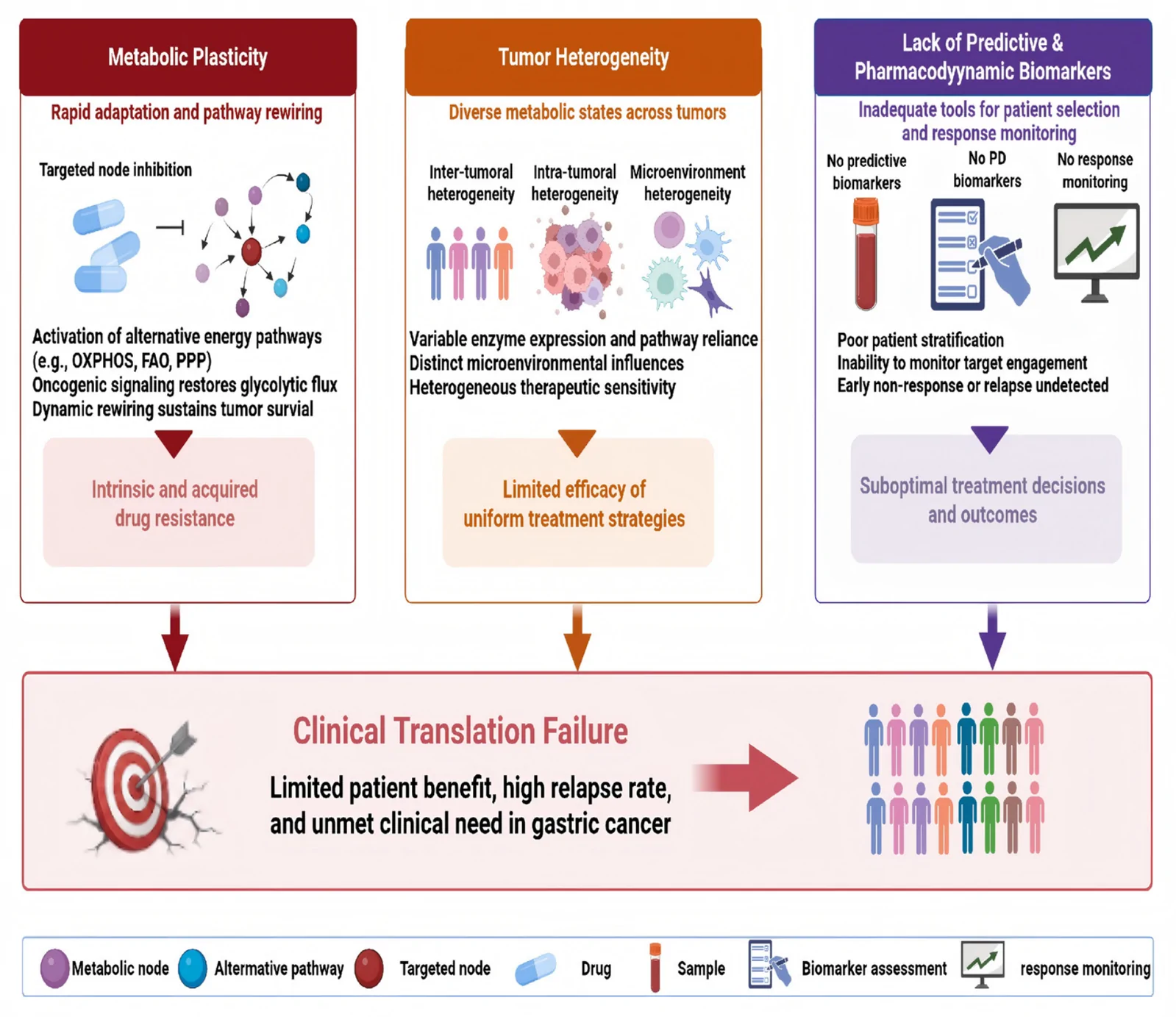
Translational roadblocks limiting the clinical success of glycolysis-targeted therapies in GC. Clinical translation is hindered by three interconnected challenges: metabolic plasticity enabling rapid pathway reprogramming, extensive tumor heterogeneity across and within tumors, and the lack of robust predictive and pharmacodynamic biomarkers. Together, these factors result in limited patient benefit and frequent therapeutic failure in clinical settings.

These roadblocks are schematically summarized in Figure 4. The following sections dissect each in turn and lay the foundation for the integrated diagnostic–therapeutic framework presented later in this review.

To provide a systematic overview of the clinical evidence supporting these translational barriers, we summarize clinically investigated glycolysis-targeting agents in Table 2. This summary is not intended as an exhaustive registry, but rather as a representative compilation of agents that have entered clinical evaluation, illustrating the recurrent challenges encountered across different metabolic targets.

Collectively, these clinical experiences indicate that the translational challenge is not simply the absence of druggable glycolytic targets, but rather the difficulty of achieving sufficient target inhibition within a heterogeneous and metabolically adaptable tumor ecosystem. These observations provide a clinical basis for the three translational roadblocks dissected in the following sections: metabolic heterogeneity (Section 4.1), adaptive plasticity (Section 4.2), and the lack of predictive and pharmacodynamic biomarkers (Section 4.3).

### 4.1. Roadblock I: Profound Metabolic Heterogeneity Across Scales

#### 4.1.1. Inter-Tumoral Heterogeneity: Metabolic Dependencies Dictated by Molecular Subtypes

The clinical translation of glycolytic inhibitors is fundamentally hampered by the profound inter-tumoral heterogeneity of GC, where metabolic dependencies are strictly dictated by divergent molecular landscapes. TCGA classification provides a robust framework for understanding these variations [101].

The Chromosomal Instability (CIN) subtype, representing approximately 50% of cases, emerges as the prototypical Warburg powerhouse. Driven by frequent amplifications of receptor tyrosine kinases (RTKs), including HER2, EGFR, and MYC, these tumors exhibit hyper-activated PI3K/AKT/mTOR signaling. This pathway activation results in robust upregulation of glucose transporters, such as GLUT1, and rate-limiting glycolytic enzymes, including HK2 and PKM2. Collectively, these alterations are supported by an expansive PPP to sustain biomass synthesis and redox homeostasis [101].

In contrast, the EBV-positive subtype displays a distinct metabolic–epigenetic interplay. While EBV-positive tumors exhibit high glycolytic activity induced by viral oncoproteins such as LMP1 and LMP2A, their metabolic program is uniquely characterized by extreme DNA hypermethylation, which can silence metabolic gatekeepers and potentially create targetable vulnerabilities absent in other subtypes.

The Microsatellite Instability (MSI) subtype—characterized by high mutational burden and robust immune infiltration—presents a different challenge: competition for glucose between malignant cells and tumor-infiltrating lymphocytes (TILs) creates a nutrient-depleted niche, where glycolytic rates are influenced as much by the immune landscape as by intrinsic oncogenic drivers [102].

Finally, the Genomically Stable (GS) subtype—often corresponding to diffuse-type GC with *CDH1* mutations or *RHOA* fusions—presents a significant therapeutic challenge due to reduced glucose dependency. GS tumors frequently exhibit a metabolic switch toward OXPHOS, glutaminolysis, and lipid reprogramming [103,104]. Recent proteomic evidence suggests that the GS subtype possesses a distinct metabolic architecture that prioritizes mitochondrial integrity over aerobic glycolysis [105].

#### 4.1.2. Intra-Tumoral Metabolic Heterogeneity: Spatial Stratification and Symbiotic Signaling

During GC progression, the metabolic landscape exhibits deep and non-random intra-tumoral heterogeneity. This topological non-uniformity originates primarily from oxygen diffusion gradient-induced metabolic polarization: in hypoxic regions of the tumor core, cells display a strong HIF-1α-dependent glycolytic phenotype; whereas in the well-vascularized invasive front, cells preferentially utilize mitochondrial OXPHOS to meet high energy demands for migration and proliferation [106]. Within this mosaic structure, cancer stem cells (CSCs) exhibit remarkable metabolic plasticity, dynamically toggling between glycolytic and OXPHOS states to sustain survival under chemoradiotherapy stress [106].

These multi-layer interactions ultimately form a metabolic symbiotic ecosystem. In this ecosystem, cells differing in spatial localization and phenotype achieve functional complementarity through metabolite exchange. This cooperation substantially reinforces the tumor’s overall resistance to stress. This system-level ecological balance has been demonstrated to be the fundamental cause of intrinsic resistance to single-pathway inhibitors in GC, highlighting the necessity of shifting future therapeutic strategies from single-target blockade to systematic disruption of the metabolic ecosystem [107].

### 4.2. Roadblock II: Metabolic Plasticity and Compensatory Resistance

#### 4.2.1. Pathway Compensation and Metabolic Switching

Cancer metabolism is inherently dynamic rather than static. Pharmacologic or genetic inhibition of glycolysis frequently triggers compensatory upregulation of mitochondrial OXPHOS, supported by alternative substrates such as glutamine or fatty acids. This adaptive metabolic switching enables tumor cells to preserve ATP homeostasis, leading to transient growth suppression followed by therapeutic escape and disease relapse [108,109]. Experimental evidence demonstrating the synergistic efficacy of combined glycolysis and OXPHOS inhibition underscores the centrality of this escape mechanism [110,111].

#### 4.2.2. Feedback Activation in Signaling Networks

Targeted metabolic interventions often elicit homeostatic feedback responses within oncogenic signaling circuits. Inhibition of the PI3K/AKT pathway, for example, can relieve negative feedback constraints on RTKs or activate parallel pathways such as MAPK signaling, thereby restoring pro-survival and pro-glycolytic signals [110,111].

A central integrator of metabolic plasticity is HIF-1α, which can be activated not only by hypoxia but also by diverse therapeutic stresses. Persistent HIF-1α signaling was identified as a key driver of immune evasion and multi-therapy resistance in GC [112], enabling broad transcriptional reprogramming that favors glycolysis, angiogenesis, and survival under adverse conditions.

#### 4.2.3. The Challenge of Enzyme Moonlighting Functions

The translational potential of metabolic targeting is further confounded by the non-canonical, or moonlighting, functions of glycolytic enzymes. As detailed in Section 2.4, these pleiotropic effects operate independently of primary catalytic roles to orchestrate malignant progression. This functional duality creates a critical pharmacological blind spot: conventional small-molecule inhibitors designed to block enzymatic pockets often leave these non-catalytic scaffolding interactions intact. Consequently, the failure of such agents to suppress moonlighting-driven oncogenic programs underscores a fundamental disconnect between metabolic inhibition and functional pathway suppression [113].

### 4.3. Roadblock III: The Critical Void in Biomarker Development

#### 4.3.1. Lack of Predictive Biomarkers

Traditional static detection at the protein or mRNA level is insufficient to predict which patients will respond to a specific glycolytic inhibitor. The dynamic, plastic nature of metabolism means static snapshots are inadequate predictors of functional pathway dependency. Emerging directions focus on functional readouts. For instance, a computational model constructed based on lactylation-related genes has been reported to assess prognosis and predict immune cell infiltration and potential response to immunotherapy in retrospective datasets [94].

#### 4.3.2. Deficiency in Pharmacodynamic and Early Response Biomarkers

A major challenge is the deficiency in pharmacodynamic biomarkers. Verifying in vivo that a glycolysis inhibitor effectively modulates its target remains difficult. ^18^F-FDG-PET measures glucose avidity. However, this modality is non-specific and may not accurately reflect therapeutic efficacy. This limitation is particularly relevant given metabolic compensation, whereby inhibition of a target pathway can upregulate alternative routes such as OXPHOS [110].

#### 4.3.3. Implications

Conducting clinical trials without robust predictive biomarkers leads to the enrollment of heterogeneous patient populations, diluting potential efficacy signals. Without pharmacodynamic biomarkers, dose optimization is empirical, and early detection of therapeutic failure is impossible.

In summary, three interrelated roadblocks—metabolic heterogeneity, adaptive plasticity, and biomarker void—collectively impede the clinical translation of glycolysis-targeted therapies. These roadblocks do not act independently. Instead, they mutually reinforce each other, creating a self-perpetuating barrier. On one hand, heterogeneity generates diverse metabolic dependencies that facilitate plastic adaptation. On the other hand, the lack of robust biomarkers hinders the selection of patients who are most likely to respond to targeted therapies. Overcoming these barriers requires a fundamental shift in both diagnostic and therapeutic paradigms. The first step toward overcoming these roadblocks is to accurately measure and quantify metabolic phenotypes in patients. Without robust diagnostic tools, patient stratification remains imprecise, and treatment monitoring is impossible. The following section focuses on translating glycolytic phenotypes into clinically actionable tools—from molecular imaging to genomic signatures and liquid biopsy.

## 5. Translating Glycolytic Phenotypes into Clinical Utility: From PET Imaging to Molecular Signatures

This section addresses this gap by focusing on how to utilize multimodal imaging, molecular signatures, and liquid biopsy technologies to translate metabolic phenotypes into clinically quantifiable monitoring tools. These diagnostic innovations are essential for patient stratification, real-time assessment of target engagement, and early detection of metabolic adaptation—prerequisites for the precision metabolic medicine framework proposed later.

Three complementary approaches are discussed: molecular imaging, genomic and metabolomic signatures, and liquid biopsy.

### 5.1. Molecular Imaging: From Metabolic Activity to Microenvironment Assessment

^18^F-FDG PET/C is long been the gold standard for assessing tumor glycolysis, but its application is undergoing a paradigm shift from a single metric to comprehensive evaluation. Clinical quantitative indicators are evolving from SUVmax, which reflects local activity, toward metabolic tumor volume (MTV) and total lesion glycolysis (TLG). Studies have shown that MTV provides information on biomass burden that goes beyond conventional anatomical size. In the CIN subtype, which is highly dependent on glycolysis, MTV/TLG are key parameters for assessing early recurrence risk and prognostic stratification, with their prognostic value surpassing that of single morphological criteria [114].

To address the challenge of false-negative fluorodeoxyglucose (FDG) findings in diffuse GC caused by low expression of glucose transporters, ^68^Ga-FAPI PET/CT targeting fibroblast activation protein (FAP) demonstrates significant advantages. It enables precise imaging of FDG-negative peritoneal metastases, achieving a leap from single-dimensional metabolism to comprehensive evaluation of the tumor–stroma microenvironment [115,116,117].

### 5.2. Genomic Stratification: Risk Prediction Based on the Glycolysis–Lactylation Axis

In the parallel dimension of imaging assessment, molecular signatures constructed by high-throughput sequencing are dedicated to decoding tumor metabolic sensitivity at the genetic level. Studies built a multi-dimensional scoring model by integrating core enzymes such as HK2, LDHA, and ENO1 along with epigenetic regulators. This model not only predicts overall survival (OS) in patients but also reveals the immune exhaustion status underlying high scores [88]. This scoring system serves as a predictive tool for identifying patients who may benefit from metabolic or immunotherapeutic interventions.

### 5.3. Liquid Biopsy: Dynamic Monitoring of Metabolic Plasticity

Liquid biopsy technologies offer unique advantages in real-time, non-invasive monitoring of metabolic plasticity. Circulating exosomes, carrying key metabolic enzymes such as PKM2, act as remote messengers of metabolic reprogramming instructions. Preclinical studies have reported that exosomal PKM2 can transfer glycolysis-dependent cisplatin resistance from resistant clones to sensitive cells and induce an immunosuppressive microenvironment by reprogramming macrophages in experimental models [118,119,120]. This makes plasma exosomal PKM2 a potential biomarker for predicting chemotherapy response and monitoring metabolic switching [121].

Looking ahead, precision monitoring is moving toward multi-omics integration. LC-MS allows monitoring of the lactate/pyruvate ratio in the peripheral blood. cfDNA methylation analysis captures epigenetic reprogramming of glycolytic genes. Together, these techniques provide multi-dimensional validation for determining whether the tumor has transitioned to metabolic escape. These integrated approaches represent the frontier of non-invasive metabolic monitoring but require further prospective validation. In summary, three complementary diagnostic modalities—molecular imaging, genomic/metabolomic signatures, and liquid biopsy—offer tools to translate glycolytic phenotypes into clinically actionable information. These approaches address the biomarker void identified above by enabling patient stratification, real-time monitoring of metabolic adaptation, and assessment of the tumor–stroma microenvironment. The diagnostic information provided by these tools is essential for implementing the precision metabolic medicine framework. With these diagnostic tools in hand, the next challenge is to design therapeutic strategies that can overcome metabolic plasticity and dismantle network resilience. The following section integrates diagnostic innovations with next-generation therapeutic modalities. The therapeutic approaches encompass dual-pathway inhibition, PROTACs, advanced drug delivery systems, and rational combination regimens. Together, these components constitute a unified framework for precision metabolic medicine.

## 6. A Strategic Framework for Solution: Integrating Diagnostics and Therapeutics

Overcoming the translational barriers associated with glycolysis-targeted therapy in GC requires a fundamental shift from reductionist, single-target strategies to an integrated, systems-level approach. As dissected in Section 4, three interrelated roadblocks—metabolic heterogeneity, adaptive plasticity, and biomarker void—consistently impede clinical success. Section 5 presented diagnostic tools to quantify these metabolic phenotypes and guide patient stratification. This section now integrates those diagnostic innovations with next-generation therapeutic modalities to form a cohesive precision metabolic medicine framework.

**Figure 5:**
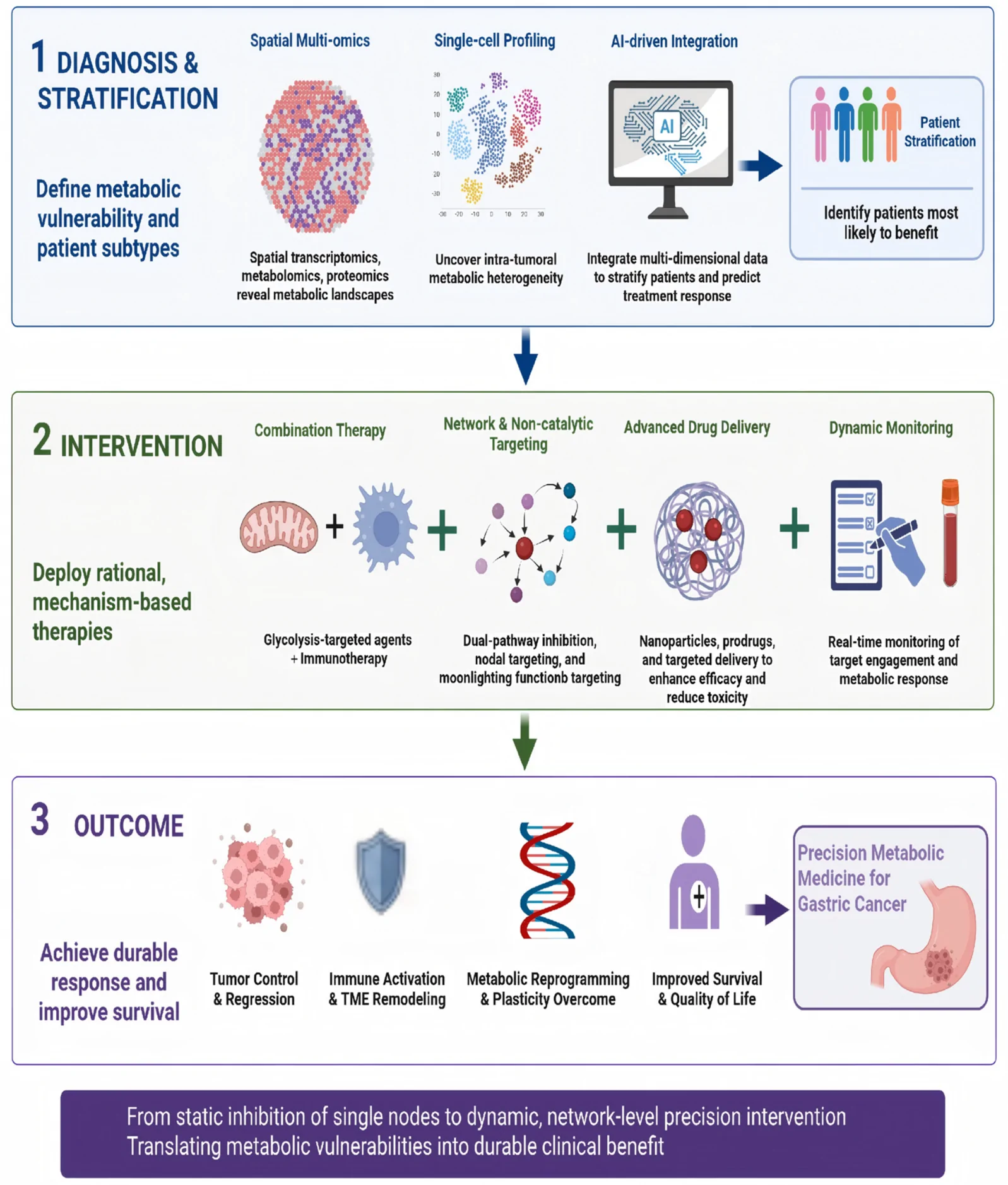
Precision metabolic medicine framework for GC. Future therapeutic strategies should integrate spatial multi-omics, single-cell profiling, AI-assisted patient stratification, and mechanism-based combination therapies to overcome metabolic heterogeneity and plasticity, ultimately enabling personalized metabolic interventions.

Three strategic pillars are discussed: diagnostic innovations to map heterogeneity and predict response, next-generation therapeutic modalities designed to overcome metabolic plasticity, and rational, mechanism-driven combination therapies (Figure 5).

While these technologies offer considerable promise, their near-term clinical implementation faces important practical constraints. Spatial multi-omics is technically demanding and costly, with limited standardization and reproducibility across centers. Serial liquid biopsy lacks unified pre-analytical protocols and requires prospective validation. AI-based integration is susceptible to dataset shift and limited interpretability. Biomarker-embedded trials increase logistical complexity and regulatory requirements. These limitations highlight that precision metabolic medicine should be advanced as an evolving translational strategy requiring stepwise implementation and rigorous external validation, rather than as an immediately deployable clinical standard.

### 6.1. Diagnostic Revolution: Mapping Heterogeneity and Predicting Response

While Section 5 focused only on specific clinical tools for quantifying metabolic phenotypes, this section addresses how emerging technologies can be integrated to resolve the complexity of metabolic heterogeneity at the systems level. Importantly, these approaches are at different stages of clinical and translational development, ranging from patient-based diagnostic and prognostic studies to emerging investigational applications that have not yet been clinically validated for therapeutic decision-making.

#### 6.1.1. Spatial Multi-Omics for Metabolic Cartography

Spatially resolved multi-omics technologies, including spatial transcriptomics integrated with proteomics and metabolomics, offer unprecedented resolution for mapping metabolic pathway activity within the architectural context of tumors. These approaches enable identification of metabolically distinct niches. These include hypoxic cores, invasive fronts, cancer stem cell reservoirs, and tumor–stroma interfaces. Such niches may harbor intrinsic or adaptive resistance to glycolytic inhibition [122]. By revealing the spatial organization of metabolic heterogeneity, spatial multi-omics provides a critical foundation for rational therapeutic targeting. However, its application to treatment selection remains primarily investigational, and prospective clinical validation of spatial metabolic profiling as a therapeutic decision-making tool is still lacking.

#### 6.1.2. AI-Driven Integrative Patient Stratification

The complexity of metabolic regulation in GC exceeds the analytical capacity of conventional univariate or rule-based approaches. Artificial intelligence (AI) and machine learning algorithms are uniquely positioned to integrate high-dimensional data from imaging, genomics, transcriptomics, metabolomics, and liquid biopsies. The objective is to define functional metabolic subtypes that more accurately predict vulnerability to specific metabolic interventions than molecular features alone [123]. Notably, the application of machine learning demonstrated high diagnostic and prognostic accuracy in metabolomics-based studies of GC [124]. However, whether AI-driven metabolic stratification can prospectively predict response to specific metabolic therapies remains investigational and has not yet been clinically validated. Extending these approaches to therapeutic stratification represents a logical and necessary next step toward precision metabolic medicine.

### 6.2. Next-Generation Therapeutic Modalities: Outsmarting Metabolic Plasticity

It should be emphasized at the outset that many of the therapeutic strategies discussed below—including dual-pathway inhibition, PROTACs, and advanced drug delivery systems—remain predominantly at the preclinical or early translational stage. Their clinical efficacy and safety, particularly in GC, remain to be established. Where available, we explicitly indicate the current stage of clinical development for each approach.

Effective therapeutic intervention must anticipate, rather than react to, the adaptive capacity of cancer metabolism. Accordingly, next-generation strategies should prioritize disruption of network stability over inhibition of individual enzymatic reactions.

#### 6.2.1. Dual-Pathway Inhibition and Modal Targeting

Preclinical studies in gastric and colorectal cancer models have demonstrated synergistic anti-tumor effects of combined glycolytic and OXPHOS inhibition, providing proof-of-concept for this approach [110]. By constraining alternative energy sources, dual-pathway targeting can induce bioenergetic crisis and limit adaptive rewiring. Nevertheless, these findings currently constitute preclinical proof-of-concept rather than clinically validated evidence, and the efficacy, safety, and therapeutic window of dual-pathway inhibition in patients with GC remain to be determined.

#### 6.2.2. Exploiting Non-Catalytic Vulnerabilities: From Moonlighting to PROTACs

As detailed in Section 2.4, glycolytic enzymes in GC exhibit moonlighting functions that drive oncogenic programs independently of their catalytic activity. This creates a pharmacological blind spot for conventional active-site inhibitors. Targeted protein degradation technologies, particularly PROTACs, represent a paradigm shift in addressing this challenge. By inducing complete elimination of target proteins through event-driven pharmacology, PROTACs can simultaneously abrogate catalytic and non-catalytic functions, thereby overcoming key limitations of conventional inhibitors [125].

Importantly, the application of targeted protein degradation is expanding beyond metabolic enzymes to include immune-regulatory proteins. This approach offers a powerful platform for integrating metabolic intervention with immunomodulation [126]. Consequently, it opens new avenues for achieving deep synergy between metabolic targeting and immunotherapy. Though PROTAC-based degraders have entered clinical development for several non-metabolic cancer targets, their application to metabolic targets in GC remains predominantly preclinical and has not yet been clinically validated.

#### 6.2.3. Advanced Drug Delivery Systems

A major obstacle in targeting glycolysis is on-target toxicity in normal tissues that rely on glucose metabolism. Nanoparticle-based delivery systems and other targeted carriers can preferentially accumulate therapeutic agents within tumors, thereby enhancing local drug concentration while sparing healthy tissues [127,128]. Such strategies not only improve therapeutic index but also enable the practical implementation of combination regimens that would otherwise be limited by systemic toxicity. However, current evidence for glycolysis-targeted nanodelivery is derived predominantly from preclinical and early translational research, and its clinical benefit, particularly in GC, remains to be established.

### 6.3. Rational Combination Therapies

For combination strategies, the evidence base varies: some rational combinations—particularly those involving immunotherapy—have begun to enter early-phase clinical evaluation, whereas others remain at the preclinical stage.

Given the intrinsic robustness of metabolic networks, rationally designed combination therapies are not optional adjuncts but essential components for achieving durable clinical responses. Three mechanistically distinct combination strategies are discussed below (Table 3).

This table summarizes three mechanistically distinct combination approaches: (1) combining glycolysis-targeted agents with immunotherapy to remodel the immunosuppressive TME; (2) combining with epigenetic therapy to disrupt the metabolic–epigenetic feedback loop; and (3) vertical/horizontal blockade targeting compensatory pathways or upstream drivers.

#### 6.3.1. Synergy with Immunotherapy: Remodeling the Suppressive TME

Glycolysis-targeted therapies are particularly well suited for combination with immune checkpoint blockade. As discussed in Section 3.1, metabolic competition and lactate production drive immunosuppression in the microenvironment. By reducing lactate production and alleviating acidosis, glycolytic inhibition can restore cytotoxic T cell function, limit immunosuppressive cell polarization, and enhance antigen presentation [19,129].

Mechanistic studies in GC provide a strong rationale for this approach. For example, in GC cell lines and mouse models, mesenchymal stem cell-mediated upregulation of PD-L1 through the CXCR2/HK2 axis has been shown to establish a link between metabolic reprogramming and immune evasion, supporting the rationale for combining HK2 inhibition with PD-1/PD-L1 blockade [130].

#### 6.3.2. Vertical and Horizontal Pathway Combinations

Vertical inhibition strategies combine glycolysis-targeted agents with inhibitors of upstream drivers, such as PI3K or HER2, to prevent pathway reactivation through feedback signaling. Horizontal combinations, in contrast, simultaneously target parallel survival pathways to induce network-wide collapse [133]. In HER2-positive GC, PGK1 expression negatively correlates with sensitivity to lapatinib. Modulating PGK1 expression affects AKT activation and cell viability. These findings support the rationale for combining glycolysis-targeted agents with anti-HER2 therapy [133]. Furthermore, disrupting the PER1-HK2 glycolysis-regulating axis was shown to reverse trastuzumab resistance in GC [134]. These findings indicate that integrating metabolic inhibitors with anti-HER2 therapy may counteract metabolic adaptations associated with targeted therapy resistance.

#### 6.3.3. Targeting the Metabolism–Epigenetics Axis

As introduced in Section 3.2, glycolytic flux in GC functions as an architect of the epigenetic landscape through histone Kla. This metabolic–epigenetic feedback loop fosters a self-reinforcing cycle of malignancy [90,131]. Pharmacological inhibition of histone lactylation, achieved either by depleting the lactate pool via LDHA inhibitors or through novel epigenetic modulators, has shown remarkable potential in enhancing the efficacy of immunotherapy in gastrointestinal cancers [132]. Combining these metabolic interventions with existing epigenetic therapies offers a synergistic opportunity to dismantle the transcriptional framework of therapy-resistant cancer, shifting the therapeutic goal from transient inhibition to fundamental cell identity reprogramming [135].

It should be noted that, with the exception of a subset of combination regimens currently undergoing early-phase clinical evaluation, most of the strategies discussed in this section remain at the preclinical stage. Their clinical applicability and safety profiles therefore require further investigation before translation into routine practice.

In summary, the proposed strategic framework integrates three complementary pillars: (1) diagnostic innovations to map heterogeneity and predict response; (2) next-generation therapeutic modalities to outsmart metabolic plasticity; and (3) rational combination therapies to dismantle network resilience. This integrated approach directly addresses the three translational roadblocks dissected in Section 4—heterogeneity, plasticity, and biomarker void—and leverages the diagnostic tools presented in Section 5. The convergence of these strategies provides a roadmap for translating the vulnerabilities inherent in the glycolytic network into durable clinical benefit. The final section summarizes the key paradigm shift and outlines actionable priorities for future research and clinical development.

## 7. Conclusions and Future Perspectives: Toward Precision Metabolic Medicine

The long and complex journey from Otto Warburg’s seminal observations to effective metabolism-targeted therapies reveals a central truth: cancer metabolism is neither static nor uniform, but profoundly adaptive and context-dependent. In GC, decades of mechanistic research firmly established metabolic reprogramming as a hallmark of malignant progression. Yet, the persistent failure to translate these insights into durable clinical benefit underscores the limitations of traditional, reductionist therapeutic paradigms.

The evidence synthesized in this review supports a fundamental paradigm shift from a 20th-century model centered on oncogene addiction and single-target inhibition to a 21st-century framework based on network addiction and dynamic, systems-level intervention. Metabolic reprogramming in GC is not a consequence of individual enzymes acting in isolation. Rather, it is maintained through interconnected metabolic circuits, feedback loops, and microenvironmental cues. Collectively, these elements give rise to three translational obstacles—metabolic heterogeneity, adaptive plasticity, and the absence of reliable biomarkers—that limit therapeutic efficacy. Therapeutic strategies that fail to account for this complexity are unlikely to achieve lasting efficacy.

To translate this conceptual shift into tangible clinical progress, three actionable priorities should guide future research and clinical development:

(1) Mandate biomarker-embedded clinical trial design. Early-phase clinical trials of metabolism-targeted therapies must incorporate mandatory biomarker programs. Predictive and pharmacodynamic biomarkers, ideally derived from functional metabolic readouts, spatial profiling, and serial liquid biopsies, are essential for patient stratification, real-time monitoring of target engagement, and early detection of adaptive resistance. Without such integration, promising metabolic interventions risk being prematurely discarded due to diluted efficacy signals in unselected patient populations [136].
(2) Prioritize biologically representative preclinical models. Preclinical evaluation of metabolic therapies should prioritize models that preserve tumor heterogeneity and microenvironmental interactions. Patient-derived organoids and advanced co-culture systems that retain stromal and immune components offer superior platforms for interrogating context-dependent metabolic vulnerabilities and optimizing rational combination strategies prior to clinical translation [137,138].
(3) Foster interdisciplinary convergence. Meaningful progress in precision metabolic medicine will require sustained interdisciplinary convergence. Integration of computational biology, artificial intelligence-driven multi-omics analysis [123], metabolic biology, immunology, and epigenetics is critical for co-developing next-generation diagnostics and therapeutics. In particular, advances at the intersection of metabolism and epigenetic regulation provide a powerful conceptual and translational framework for disrupting self-reinforcing oncogenic circuits [139].

Several limitations of this review should be acknowledged. First, our literature search was restricted to English-language publications indexed in PubMed, which may have introduced language and publication bias. Second, the evidence discussed is drawn predominantly from preclinical mechanistic studies and early-phase clinical trials, whereas large-scale prospective validation of glycolysis-targeted strategies in GC remains limited. Third, heterogeneity in study designs, patient populations, and biomarker assessment methods across the included literature limits the ability to perform quantitative synthesis or draw definitive conclusions regarding clinical utility. Fourth, although we discuss emerging technologies such as spatial multi-omics and AI-based approaches, their current evidence base remains primarily exploratory, and prospective validation data are still lacking. These limitations highlight the need for continued investigation and cautious interpretation of the findings presented in this review.

In conclusion, the field is now equipped to address the translational challenges that have long hindered glycolysis-targeted therapy in GC. This is achieved through an integrated, challenge-focused, and solution-oriented framework. The framework encompasses diagnostic innovation, next-generation therapeutic modalities, and rational combination strategies. The molecular understanding is deep, the technological toolkit is rapidly expanding, and the clinical need remains urgent. The opportunity now lies not in discovering new metabolic targets, but in deploying existing knowledge with strategic precision to deliver durable, metabolism-informed therapies for patients with GC.

## Figures and Tables

**Table 1:** Key glycolytic effector enzymes: regulation, function, and clinical significance.

Enzyme	Regulation & Oncogenic Role	Clinical Significance
HK2	PI3K/AKT activates; NLRP12 [21], SALL4 [22]; anti-apoptotic via VDAC binding; prevents Bax mitochondrial translocation	Associated with advanced TNM stage, metastasis, chemoresistance, and poor PFS in cohort studies [23]
HKDC1	*H. pylori*-induced TGF-β/Smad2 [24]; promotes EMT, metastasis [25]	Identified as an independent poor prognosis factor in clinic cohorts; with diagnostic in *H. pylori +* diffuse-type GC [24]
PKM2	Rab25 [26], KAT2A [27] regulate; dimer form shunts to PPP; nuclear PKM2 activates β-catenin/STAT3 [28]	Linked to proliferation, EMT, and 5-FU resistance in preclinical and clinical studies [29]
LDHA	HIF-1α, c-Myc activate; maintains high glycolytic flux; promotes M2 macrophage polarization and PD-L1 upregulation via lactate [29]	Associated with poor prognosis in GC patients; preclinical evidence links M2 polarization, PD-L1 upregulation [30,31]
ENO1	AKT, ncRNAs, SUMOylation regulate [32,33,34]; promotes proliferation, metastasis, stemness	Associated with cisplatin resistance and aggressive tumor behavior in experimental models [21,32]
PGK1	TRIM8 stabilizes [35], TRIM50 degrades [36]; mitochondrial PGK1 enhances Warburg effect [37]	Linked to anoikis resistance in GC cell line [38]
PDK4	Transcriptionally activated by HIF-1α/c-Myc; inhibits TCA cycle, diverts pyruvate toward lactate synthesis [39]	Associated with metabolic reprogramming [39]
ALDOB	Tumor suppressor; downregulation enhances glycolysis via PI3K/AKT [40]	Downregulation correlates with poor prognosis [41]

**Table 2:** Clinically investigated glycolysis-targeting agents in cancer: clinical development, outcomes, and translational barriers.

Graph	Agent	Clinical Phase	Cancer Type	Major Clinical Outcomes	Major Translational Barriers/Reasons for Limited Success
HK2/glycolytic flux	2-Deoxy-D-glucose (2-DG)	Phase I/II	Advanced solid tumors, lung, breast, pancreatic, head and neck, gastric, castration-resistant prostate cancer	Dose escalation established feasibility and recommended Phase II dose (45 mg/kg). A separate Phase I/II study in advanced cancer was terminated because of slow accrual and failure to demonstrate single-agent activity [94].	Dose-limiting toxicity and narrow therapeutic window; insufficient single-agent antitumor efficacy; metabolic compensation; difficulty translating glucose uptake inhibition into durable tumor control.
PFKFB3	PFK-158 (ACT-PFK-158)	Phase I	Advanced/refractory solid malignancies	First-in-human Phase I dose-escalation study demonstrated acceptable tolerability and pharmacodynamic modulation of F2,6BP and FDG-PET. One patient showed a marked reduction in hepatic metastatic burden, but robust objective efficacy was not established [95,96].	Limited clinical efficacy despite target inhibition; lack of validated predictive biomarkers; heterogeneous tumor metabolism; adaptive metabolic responses may compensate for glycolytic suppression.
Mitochondrial hexokinase/OXPHOS- glycolysis coupling	Lonidamine	Phase II	Glioma, renal cell carcinoma, breast, ovarian, head and neck, lung cancer	First-in-human Phase I dose-escalation study demonstrated acceptable tolerability and pharmacodynamic modulation of F2,6BP and FDG-PET. One patient showed a marked reduction in hepatic metastatic burden, but robust objective efficacy was not established [97,98].	Modest and inconsistent efficacy; gastrointestinal/muscular toxicity; lack of tumor-selective metabolic dependence; insufficient efficacy to support development as a stand-alone glycolysis-targeted therapy.
PDK1/2/3 (pyruvate dehydrogenase kinases)	Dichloroacetate (DCA)	Phase I; Phase II/IIA	Advanced solid tumors; glioblastoma	Phase I study (*n* = 24) characterized safety and PK, and established a recommended Phase II dose. Extended to recurrent glioblastoma, including a Phase IIA surgical study designed to assess pharmacodynamic modulation of PDH phosphorylation [99].	Neurologic toxicity and dose limitations; uncertain efficacy across heterogeneous tumor types; dependence on intact PDH biology and tumor metabolic context; inadequate predictive biomarkers.
LDHA/BCL-2 family	Gossypol/AT-101	Phase I/II	Advanced solid tumors; breast cancer; multiple myeloma; small-cell lung cancer	Early clinical studies showed limited antitumor activity. In refractory metastatic breast cancer, only one minor response and several stable-disease cases were observed; dose-limiting toxicity occurred at higher doses. Earlier studies reported no objective tumor regressions among evaluable patients [100].	Limited objective efficacy and substantial toxicity; poor target specificity (affects multiple metabolic and non-metabolic pathways); insufficient therapeutic window for effective glycolytic blockade.

**Table 3:** Rational combination strategies targeting metabolic reprogramming in GC.

Combination Strategy	Key Rationale	Examples	References
Immunotherapy	Reduces lactate production, reverses immunosuppression, restores T/NK cell function	LDHA inhibitor + PD-1/PD-L1 antibody; HK2 inhibitor + PD-1 antibody	[19,129,130]
Epigenetic Therapy	Blocks lactate-driven histone lactylation, disrupts metabolic–epigenetic feedback loop	LDHA inhibitor + epigenetic drug	[90,131,132]
Vertical/Horizontal Blockade	Targets compensatory pathways or upstream drivers HER2	Glycolysis inhibitor + OXPHOS inhibitor; Glycolysis inhibitor + HER2 inhibitor	[110,133,134]

## Data Availability

No new data were created in this study.

## Abbreviations

The following abbreviations are used in this manuscript:

ALDOA	Aldolase A
ALDOB	Aldolase B
AI	Artificial intelligence
CA IX	Carbonic anhydrase IX
ceRNA	Competing endogenous RNA
circRNA	Circular RNA
CSCs	Cancer stem cells
EMT	Epithelial–mesenchymal transition
ENO1	Enolase 1
FAP	Fibroblast activation protein
FDG	Fluorodeoxyglucose
GAPDH	Glyceraldehyde-3-Phosphate dehydrogenase
GC	Gastric cancer
GLUT1	Glucose transporter 1
GS	Genomically stable
*H. pylori*	*Helicobacter pylori*
HER2	Human epidermal growth factor receptor 2
HIF-1α	Hypoxia-Inducible factor 1-Alpha
HK2	Hexokinase 2
HKDC1	Hexokinase domain containing 1
HKs	Hexokinases
HREs	Hypoxia response elements
IGF2BP2	Insulin-Like growth factor 2 mRNA-binding protein 2
LDHA	Lactate dehydrogenase A
lncRNAs	Long Non-Coding RNAs
m^6^A	N^6^-Methyladenosine
MSI	Microsatellite instability
MTV	Metabolic tumor volume
ncRNAs	Non-Coding RNAs
NLS	Nuclear localization sequence
OS	Overall survival
OXPHOS	Oxidative phosphorylation
PDH	Pyruvate dehydrogenase
PDKs	Pyruvate dehydrogenase kinases
PDK1	Pyruvate dehydrogenase kinase 1
PEP	Phosphoenolpyruvate
PFS	Progression-Free survival
PGK1	Phosphoglycerate kinase 1
PKM2	Pyruvate kinase M2
PPP	Pentose phosphate pathway
PPIs	Protein–Protein interactions
PROTAC	Proteolysis-Targeting chimeras
PTMs	Post-Translational modifications
RTKs	Receptor tyrosine kinases
SALL4	Spalt-like transcription factor 4
TAMs	Tumor-Associated macrophages
TILs	Tumor-Infiltrating lymphocytes
TLG	Total lesion glycolysis
TME	Tumor microenvironment
VDAC	Voltage-dependent anion channel
